# Living with Chronic Pain: A Qualitative Study of the Daily Life of Older People with Chronic Pain in Chile

**DOI:** 10.1155/2019/8148652

**Published:** 2019-04-01

**Authors:** Iyubanit Rodríguez, Esmeralda Abarca, Valeria Herskovic, Mauricio Campos

**Affiliations:** ^1^Department of Business Computer Science, Universidad de Costa Rica, Alajuela, Costa Rica; ^2^School of Nursing, Pontificia Universidad Católica de Chile, Santiago, Chile; ^3^Department of Computer Science, Pontificia Universidad Católica de Chile, Santiago, Chile; ^4^School of Medicine, Pontificia Universidad Católica de Chile, Santiago, Chile

## Abstract

One of the main causes of chronic pain in older people is spine deformity, an abnormal curvature of the spine. The purpose of this study is to improve understanding of the experience of chronic pain produced by spinal deformity in older people and understand how cultural factors may affect this experience. A qualitative study was performed with 10 older people. Participants were encouraged to describe a typical day in their life, including the factors that affect their pain and how their quality of life had been affected since experiencing chronic pain. The semistructured interviews were transcribed and analyzed using open coding. Pain caused by spine deformity produces disability, affecting how older people perform their daily activities, causing them to adapt their households and physical positions to perform these tasks, albeit slowly or incompletely. Chronic pain also affects emotional states and social relationships because older people become unable to undertake physical activities that they performed in the past. The close relationship with adult children and friends, typical in Latin cultures, is a source of comfort and support. At a community level, adaptation of public services (such as public transportation) must be improved.

## 1. Introduction

Chronic pain is pain that lasts more than 3–6 months that adversely impacts the well-being of the affected individuals [1], limiting and reducing their physical activity, social relations, and energy levels [2]. When pain is widespread, it is common for people to regard their body as a crippling obstacle [3], resulting in them having to change their normal lives while simultaneously harbouring feelings of helplessness and vulnerability [4]. They experience shame and fear of rejection and feel as if they are a burden to others [5]. In turn, this leads to feelings of isolation and a fear of the future [6]. Among the four main causes of pain are spinal issues [7], e.g., those caused by the curvature of the spine in older adults [8]. Spine deformity includes asymptomatic curves and progressive or disabling deformities [9]. Spine imbalance requires a high amount of energy to try to maintain balance, causing people to become fatigued and feel pain [10, 11]. The deformity also produces feelings of insecurity, lack of attractiveness, depression, and social isolation [12].

Pain interferes with the daily activities of people and disrupts every facet of their lives, causing a range of frustrations that stem from the invisibility of the pain and associated limitations in the diagnosis and treatment thereof [13]. Chronic pain also has serious detrimental effects on the social and familial environment of people. As a consequence, a multidisciplinary approach to treatment that includes the family and social context is required [14]. Furthermore, understanding the subtleties of the experience of pain may help health professionals to devise diagnoses and treatments that are more suited to the characteristics of each particular population. Thus, additional research is required into populations with different ethnic backgrounds to assess how ethnicity and culture can influence the experience of chronic pain [15]. Likewise, studies have suggested the existence of ethnic-based differences based on the threshold and intensity of pain and their impact on patients [16, 17]. Additional research that makes reliable comparisons between Latin American countries and other regions is needed [18]. This work provides insight into the experience of Chilean older adults with chronic pain, describing through a qualitative study how pain affects their daily lives and their wellbeing and explaining our findings through the Chilean cultural and societal context.

### 1.1. The Chilean Context: Older People

In Chile, approximately 30.6% of its population will be over 60 years old by 2050 [19]. Pain caused by the back, knees, hips, and other joints is the most commonly cited health problem among older people in Chile [20]. In this section, we briefly describe how older people live in Chile. Chilean society is based on social links built around primary relationships [21], in which goals and interests of the group take precedence over those of the individual [21]. The burden of managing Chilean families is primarily borne by women, especially in terms of household chores and care-related tasks, whereas men are traditionally viewed as the financial providers [22]. It is common for two or three generations to live under one roof; for example, 59% of older people live with one of their children and contact with offspring who do not live in the same house as their parents is regular [20], usually taking the form of a monthly family meeting [23]. These intergenerational relationships work in both directions in terms of emotional support, advice, and practical help, such as caring for a grandchild [20], which 35.7% of older people in Chile do at least once a week [20]. Within the Chilean family, older people occupy a position of respect [21].

In relationships between older people and their adult children, there is a filial obligation that consists of the provision of economic and emotional support to the former during their old age. From the point of view of the child, this relationship is seen as a way of repaying the past debt of moral education and dedication bestowed upon them by their parent(s), whether or not they live in the same home in the present [23]. Nevertheless, older people in Chile have shown limited support for this type of obligation [20]. In general, older people continue to actively perform their own personal and household chores as part of their desire not to become overly dependent on others [23], which is one of the most common concerns of people within this age group in Chile [20]. However, older people often turn to their family, especially their children, for needs relating to, for example, transportation to the hospital or assistance in terms of their essential daily activities [20, 23].

### 1.2. Chronic Pain in Chile

Non-oncological chronic pain is estimated to occur in 32% of the adult population in Chile, and approximately 70% receives some drug treatment for chronic pain [24]. The level of chronic pain is divided into the following: mild (11.8%), moderate (65.7%), and severe (20.8%) [24]. Chronic pain is more frequent in women and in older people [25]. It has been mentioned that chronic pain is an important cause of disability and is responsible for a high social and financial burden in Chile, since the consequences associated with chronic pain are expensive for health systems and for society in general [26].

In Chile, there is a National Program of Pain Relief and Palliative Care of the Ministry of Health, which is intended for cancer patients. Through this program, units focusing on pain have opened up both in private and public healthcare centers, which now also deal with other types of pain. Regarding pain management in Chile, several issues have been described, e.g., the long duration of symptoms, insufficient response to medical treatment, high use of anti-inflammatory drugs, and low use of specialized services in pain management [24].

Therefore, the objective of this work is to generate a more comprehensive understanding of the experience of pain in the daily lives of older people with spine deformity in Chile. Semistructured interviews were conducted with ten older people who endure chronic pain, with the objective of understanding their experiences of living with this condition in the Chilean context.

## 2. Materials and Methods

The study was exploratory, descriptive, and comprehensive, using a qualitative methodology, thereby enabling the reader to comprehend real-life experiences from the perspective of the participants [27]. We conducted semistructured interviews. The interview is the most common format for data collection in qualitative research [28]. Semistructured interviews are those where participants have to answer open questions [28]. These types of interviews are carried out only once, with an individual or with a group [29]. These interviews are based on a guide, which is a schema with questions or topics that serve the purpose of exploring many participants in a systematic and exhaustive way, as well as to keep the interview focused on the objective [29].

We used a 32-item checklist for qualitative studies to report this work [30]. This approach was used to understand the perception of people with spine deformity in regard to how they live with chronic pain, from a Chilean cultural perspective.

### 2.1. Data Collection

For this study, older people with chronic pain and spine deformity were recruited from a university hospital in Santiago, Chile, to participate in a semistructured interview. The interviews were conducted in Spanish by one (native Spanish-speaking) researcher during a period of 4 months in a location selected by the participant: either the hospital, a coffee shop or their own home. There was no pilot testing. Interviews were semistructured; participants were encouraged to describe a typical day in their life, including the factors that affect their pain and how their life had changed since experiencing chronic pain and spine deformity. Each interview lasted 30–60 minutes and was audiotaped and transcribed verbatim. Following the interview, participants were asked to complete three questionnaires with the objective of obtaining demographic data:

- Oswestry Disability Index (ODI) is a self-administered questionnaire used to evaluate limitation of activities regarding daily life [31].
- Scoliosis Research Society-22r patient questionnaire evaluates the effect of idiopathic scoliosis and its treatment from the perspective of the patient in five domains [32]. There are 22 questions, and the questionnaire is scored so that 1 is the worst response and 5 the best [33].
- World Health Organization Quality of Life (WHOQOL-BREF) survey assesses the perception of individuals in relation to their culture, value systems, and personal goals and concerns [34].

These questionnaires were selected because they are validated in their respective field and can give us information of the participants about their quality of life, their disability, and their deformity, which allow us to have a better profile of the group of participants.

### 2.2. Participants and Data

Orthopaedic surgeons from the university hospital in which participants were recruited passed the relevant study information to the individuals involved. If they decided to subsequently take part, the first author contacted them to arrange the details of their participation. The inclusion criteria for participation were as follows: aged over 65, Spanish-speaking, resident in Chile, and experiencing spine deformity, and pain for at least six months. People with hearing or speech problems, as well as illiterate individuals (because the participant must fill out questionnaires), were excluded from participating due to the nature of the study.

The participants were eight women and two men, ranging in age from 67 to 79 (md = 73, SD = 4.13). Five of them were housewives, three were employed, and two retired. The participants had experienced chronic pain for an average of 5.1 years (min. = 1 year, max. = 18 years); six of them smoked (md = 21.5 years), one was a passive smoker, one did not smoke, and two chose not to answer the question about whether they were a smoker. The quality of life of the participants, according to the WHOQOL-BREF questionnaire, indicates that physical health and social relationships were the lowest scoring dimensions in this group.

Table 1 displays age, gender, spinal issue, and ODI and SRS-22r scores for all study participants. According to the ODI questionnaire, there are 5 levels of disability [35]:

1. 0%–20% (minimum disability): the patient can perform most of life's activities
2. 21%–40% (moderate disability): the patient experiences more pain and difficulty when sitting, lifting objects, and standing
3. 41%–60% (severe disability): pain remains the main problem in this group and daily activities are affected
4. 61%–80% (crippled): back pain affects all aspects of the patient's life
5. 81%–100%: these patients are bedridden or exaggerating their symptoms

The level of disability was minimal for two participants, moderate for four, severe for three, and exaggerated for one person.

### 2.3. Data Analysis

Data analysis was conducted in Spanish (language of the participants and researchers). Open codification (based on the grounded theory methodology) was used for data analysis, since the aim of the study was to describe and, therefore, understand the perspective of participants [36]. We used an inductive process and analytical operations geared towards answering our question. Subsequently, each single interview extract was compared with additional extracts in order to identify any similarities and differences. This analysis generated codes that were grouped into subcategories until the main ones were obtained [36]. The Atlas.ti program was used to facilitate this process (https://atlasti.com).

To ensure the methodological accuracy of the results, a triangulation strategy was used [37], which involved the participation of two researchers from different areas of study: nursing and computing; for which there were different points of view when analyzing the data. This helped to give rise to an intersubjective agreement on the generated categories. Both researchers jointly codified two randomly selected interviews, creating a list of initial codes. Thereafter, the remaining eight interviews were shared between the two authors, who codified them individually using the initial codes. Regular meetings were held to assess agreement on the coding and analysis and improve or modify the generated codes iteratively. Data saturation was reached at the sixth interview. Transcripts were subsequently re-read several times, and themes were identified by means of a process of repetitive interpretation, synthesizing, and theorizing. Finally, a consensus was reached on the generated categories.

### 2.4. Ethical Considerations

The study protocol was approved by the university ethics committee (15-339). Prior to the interviews being conducted, participants received oral and written information about the aim of the research, while written consent to the overall study was subsequently provided by each individual. Participants were informed that their involvement was voluntary, that anonymity would be guaranteed, and that they could withdraw from the study at any time.

## 3. Results

**Figure 1:**
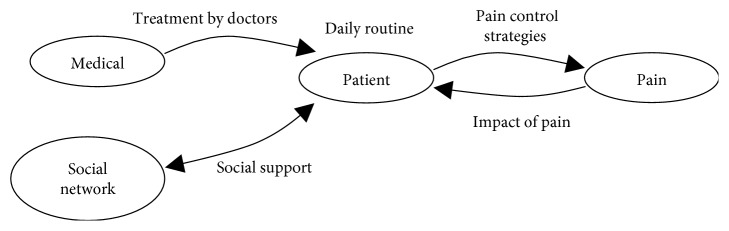
Descriptive analysis: a diagram of the main categories.

Following the descriptive analysis of the 10 semistructured interviews with older people with chronic pain and spine deformity, five main categories emerged: impact of pain on the patient, pain control strategies, treatment by the doctors, daily routine, and social support. The model (Figure 1) is patient-centered, since only older people with chronic pain were interviewed; therefore, all categories are related to the patient. The doctor-patient relationship is the patient's perception regarding his/her treatment. Pain is related to the patient in two ways: (1) how the pain impacts the person (arrow from pain to patient); (2) how the person controls the pain he/she feels (arrow from patient to pain). Finally, the person's social network is affected by the disease, and their support (or lack thereof) also affects the older people. This section explains these categories. Quotes taken directly from the interviews and translated from Spanish to English are provided below.

### 3.1. Daily Routine

Participants were asked about a typical day in their lives, including the main obstacles that they have to overcome and the factors (environmental or psychological) that affect the intensity of their pain.

In the morning, participants get up and out of their beds unaided before taking their medicine and having breakfast. They then wash. They explained how they had refurbished the bathroom in order to be able to wash and bathe more securely and without help. To provide brief context, it should be noted that it is common for the shower to be set within the bathtub in Chile.

I now realize that I could fall over at any time, that this or that could happen to me. I made some changes to my bathroom, took the bath out, installed a shower space, which is about this high, so I don't have to lift my legs much to get in, meaning that I won't stumble. I've got handrails, in the bathroom, to hold on to, but I don't wash standing up in the shower because it's a nightmare for me, which is why I've got a special seat, like a special stool, and I use the showerhead to splash myself with water. I use the soap to wash myself, but it takes me a long time.

After washing, participants get dressed. Some do this slowly albeit on their own, while others require help, for example, from their children:

It takes me a long time, but I can do it. With the bra, I can't do it up from the back. I put it on from the front and pull it up. To undo it, I turn it around because I can't do that normally either.

The majority of participants do not work and therefore undertake domestic chores after the completion of their personal hygiene routines. A common theme that arose in the accounts of the participants is whether or not they receive assistance in performing these domestic chores. In particular, this included whether or not they employ a nana (housekeeper) to assist them in certain domestic tasks.

I find it difficult to make the bed, I... I simply tidy it up. Later, when my daughter or the nana come, they make it properly. I just tidy it up, I don't make it properly. I mean, I do it lazily. Afterwards, when I have to run an errand, pay the bills, or go to the doctors, I go out, and a young girl comes (to help out in the house) once a fortnight. I keep some areas clean, where I can, and when the young girl comes, she does a more thorough clean.

In the evenings, the participants rest or carry out light activities, such as watching television, sharing time with grandchildren, knitting or, in some cases, attending physiotherapy sessions or medical appointments. Furthermore, none of the interviewees are responsible for caring for their grandchildren. Rather, they mentioned that they simply visit them on a regular basis and frequently accompany them on daily activities.

After lunch, I walk to my son's house, which is nearby, about one block in fact. There I take care of my grandson, with the help of the nana too. I just accompany them really. I rest a bit with him, and we go for a walk at about half past four. We walk for an hour, or an hour and ten minutes. Slowly, but we walk for an hour and ten minutes. That's what we do every day.

Finally, participants go to sleep, but in some cases, they wake up at night, because they feel pain.

No, I can't sleep very well. I used to sleep soundly […] I think all of this is the result of all the traumas I've been having, health wise…

### 3.2. Impact of Pain on Older People

Pain produces disability and affects the way in which the participants are able to perform their daily activities. The majority can sit without problems, but they find other activities (walking, standing, and lifting items) more difficult.

Sometimes, to go to the bathroom, I can't even get there... especially when I have to hold myself up, I wake up wanting to cry because I think I'm going to fall [...] it's because I find it a real effort to stand up.

Chronic pain affects the emotional state of the participants involved in this study. Each female interviewee claimed to have (or have had) symptoms of depression (e.g., anhedonia). They suffer so severely from depression that they feel like another person entirely.

I feel really downcast, with no desire to do anything, because to top it all I have depression [...] I used to be really active, and now I'm not the same person.

Despite a number of participants using medicine for their depression, the majority of those who experience bouts of depression receive no medical attention for their symptoms. For example, they mentioned that rather than seeking out medical assistance, have sought support in spirituality via prayer:

No, none. What greatly helps me are the (Catholic) saints. I pray to our Lady of Guadalupe, to the Virgin Mary... I pray to all my saints, always.

The majority of participants feel that their social life has been limited and reduced by their pain. Two of the reasons for this are that they are unable to undertake the physical activities that they performed in the past and that they feel that other people are unable to empathize with them.

For two reasons: because I feel diminished and because people diminish you. They don't pay attention to you. It's as if you are just left out of certain things. I have a friend here in Santiago, and she likes walking even more than I do. She likes going out. And so, I do... but I can't keep up with her. She makes me walk fast. Only once did she do this, and now I don't go out with her anymore.

The primary means of public transportation in Santiago is via the subway system or bus service. During their interviews, the participants indicated that these forms of transport were problematic. For example, the movement of the buses increases their levels of pain; the drivers close the doors too quickly when passengers are trying to disembark the bus; and most subway journeys have to be undertaken standing up, causing additional pain. In addition, accessibility is poor, for example, a number of subway stations lack facilities for people with physical disabilities:

When I [...] return home, there is no elevator in the closest station to where I live. There is no escalator either, so I go up the stairs slowly and I get to the top feeling exhausted, as though I have no more energy left.

However, this category presented dichotomous elements, since other participants mentioned that pain does not affect them as much, for example, regarding sleep or mood.

Not at all. It's part of life.

### 3.3. Social Support

The participants were satisfied with the social support they receive from their friends. These findings suggest that, although people have decreased their social activity, they still feel that their friends offer them support. This could be because friends maintain contact and provide support to them. For example, they mentioned that despite not being able to leave the house, their friends visited them at home:

I have a group of friends and we all got together on Friday mornings to have coffee in J^*∗∗*^ coffee house, and the best part is, well... now, they come and see me. But like I said, it's not the same anymore.

Most of the participants live with their adult children. The additional household members provide help with transportation and chores and take care of them.

My daughter calls me regularly. My son too, and they look after me well. I'm not alone because I'm with them.

However, some of the participants feel that their family members do not fully understand their disease. Specifically, this problem stems from the limited communication that exists between members of the family, stating that these kinds of issues are not discussed in the household.

I feel like I have no family support, and that communication with the family—especially with the people who I live with on a daily basis—is a bit complicated because they work. So, in terms of communication, they often get home from work and do this, that or the other, and then simply go to bed... so communication is really lacking. If there were more communication, maybe the family environment would be more enjoyable.

They felt they were bothersome to their family and that their family no longer took their seriously. This causes them to downplay things and withhold their true feelings.

... because I get the feeling that they think that I'm doing it to myself, that it's a way of trying to draw attention to myself because I feel bad, in pain. I express it in the way I want to, and sometimes I have to hold back...

### 3.4. Treatment by Doctors

Regarding medical support, the participants are satisfied with the treatment received to date and they would return to receive the same treatment again. The majority of the participants have sufficient information and understanding about their illness and treatment, to the extent that some of them were able to describe their medical conditions in detail.

It's one of the things that caught my attention: the doctor was always very attentive. I mean, the doctor didn't just ask questions in passing; he gave me his diagnosis and information about what to do at home. He was always very attentive and that is why I trusted him. I felt comfortable, and I still feel comfortable with him.

### 3.5. Pain Control Strategies

It was difficult for participants to describe their pain. Rather, they recalled factors that contribute to its increase or decrease, or the feelings that it induces.

It's so difficult […] it's hard to explain... How do I explain it? On one hand, it's tough to explain, and on the other, understanding the pain is difficult, because someone else would have to feel it. Even my daughter told me it can't hurt like that... well, but it can't be like that, but it hurts, what do you want me to do? You don't have a broken back. It's broken, what can I say... I didn't make it up... look at my X-ray...

In order to carry out their daily activities, participants frequently adopt certain actions or physical positions that help to mitigate their pain. These actions and positions have been borne out of the personal experience of each participant and were not taught by a health professional. As such, this behaviour should be understood of as a reaction and/or way to simply get through the day. For example, they described the position that they adopt in order to do the washing up: “*I stand up and go to the sink, walking with my legs apart, and straight, in line with my shoulders... that way I don't feel any pain*”. Another form of mitigating pain is to take short breaks, such as sitting or lying down: “*even when I'm cooking, sometimes I have to lean on something because I'm in so much pain, and I have to go and lie down in bed for a while, rest for about ten minutes and then I get back up and carry on with my chores.*”.

The treatment received by participants in Chile with symptoms such as those experienced by the participants of this study includes medicine and/or physiotherapy sessions. Some participants use painkillers and the majority require this type of drug in order to be able to go about their daily lives.

Some participants additionally use alternative therapies to reduce their pain:

The most effective thing for me has been acupuncture. It has helped me quite a lot. It doesn't make the pain go away completely but it does lessen it. But this approach involves commitment and I'm going to keep it up. I asked the doctor about it and he said yes, absolutely. [...] Among things that are not medical-based, acupuncture is what I like the most, and it's paying off.

Participants mentioned that they have trouble sleeping at night and that in order to mitigate their pain, they have to find a physical position that allows them to go to sleep, or they take sleeping pills.

Well, I've always slept badly. It's difficult for me. I have suffered from insomnia in the past, but now it's more because if I roll over in bed I have to hold on to the headboard in order to move, and by then I have woken up. I find nights hard... they're difficult for me.

In spite of the difficulties of living with this disease, the participants interviewed in this study attempt to be positive and remain active. For example, “*I try not to spend too long lying down. I try to walk, to anywhere I can. I walk to the kitchen, I try to do other things too... things that I can manage*”. Likewise, they employ a range of strategies to control their emotional state:

I'm not saying that I don't get down sometimes, because I'm not perfect. [...] If I'm feeling a bit down, like I don't want to get up, I get up anyway. I don't let it overcome me. Sometimes I can't even be bothered to cook for myself, but I still make something all the same. I took a little course on controlling my mental state and I'm using what I learned now. I never used to use it, it always seemed pointless to me... but now I find it really useful.

## 4. Discussion

Chronic pain is a complex phenomenon, composed of dichotomous categories and characterized by multiple components that modify and/or alter its manifestation and increase or decrease its intensity. These results are comparable to previous related research [6, 15, 38]. Older people in this study described their pain experience as incapacitating and, therefore, life changing, impeding the execution of daily activities and requiring rest after their completion. In previous studies, older adults with vertebral deformities had functional limitations and difficulty with activities of daily living [39]. Older people in Chile, however, do not want to depend on other people, so they try to do their personal grooming and housekeeping activities, although they do slowly or incompletely, sometimes requiring assistance anyway.

A further area of difficulty faced by the participants in their daily lives is public transportation; this restricts their ability to leave their homes to undertake personal tasks and attend doctor's appointments or social activities. Chilean seniors like to communicate face to face or meet with members of their peer group in person, but problems in public transportation minimize the possibility that they can carry out this type of social interaction. This finding is particular to the location of our research, as many subway stations do not have elevators, some do not have escalators, and buses generally have a few high steps. Evidence of similar issues have been found in other developing countries, e.g., older women with vertebral fractures chose to use private, rather than public, transportation [12].

Chronic pain has further repercussions on personal experiences related to emotional well-being and social relationships. With respect to social relations, participants are prevented from attending social events or are limited to making plans in advance due to the unpredictable nature of the disease [14]. Therefore, the social life of participants living with chronic pain is limited or reduced. However, our study shows general satisfaction in regard to the support received from friends, despite indications that their social relations have changed since they began to experience chronic pain. Older people in Chile usually prefer to meet members of their peer group in person, for recreational activities, mutual support, and general company, as well as to share common experiences [40]. The aforementioned is due to the close relationships that they culturally have with friends and families. In our study, participants felt that friends and family are in contact with them, either through home visits or phone calls, which generates a feeling of support. The cultural obligation for families to provide care for older people, combined by the participants' feelings that others are not empathetic towards their condition, causes some tension in family relationships. This suggests that treatment of these people should include their families, in order for them to understand the complexity of this disease.

Regarding emotional well-being, the two participants who said that they had experienced no bouts or symptoms of depression as a result of their back pain were men. In contrast, the remaining eight participants, all of whom were women, indicated that they had either had or still have certain symptoms of or, indeed, diagnosed, depression. These findings coincide with evidence provided by a Canadian investigation in which people with chronic pain and who experience major depression are primarily older women [41]. In Chilean culture, women traditionally have the responsibility to manage household chores and care-related tasks, so not being able to fulfil them causes frustration, stress, and depression.

Satisfaction with medical treatment and the trust of participants in their physicians is stated in interview testimony. The way in which participants perceive health care professionals, in regard to their availability, explanation of treatment, and susceptibility to the feelings of the affected individual, can be an important factor in their trust of the treatment received. Practitioners who try to form warm and friendly relations with their patients show more positive results in terms of patient health than practitioners who conduct their consultations in an impersonal, formal, or uncertain manner [42]. There is evidence of patient satisfaction with doctors who are sensitive to their experience [43], and the inverse has also been reported [15, 44]. Participants in our study were treated by only one team of physicians with a similar demeanor and training, which explains the participant' satisfaction with their treatment.

Faced with their chronic pain and spine deformity, the study participants stated that they try to retain a positive mental attitude and have developed coping strategies to mitigate the pain in the daily lives. These strategies include adopting antalgic positions, utilizing a range of devices, avoiding the execution of certain actions, taking pain medicine, and requesting assistance from others. Therefore, they have adapted to their new reality of living with a spine condition, particularly in relation to the chores and activities that they are able to carry out. It should be noted that the coping strategies of our participants have arisen from the basis of their understanding of their condition and their personal experiences; according to their interviews, the coping strategies were not taught or provided by health care professionals.

## 5. Conclusions

This study found that pain caused by spine deformity is a unique, personal, and subjective experience with no clear or specific definition. As a consequence, it is difficult for the individuals who experience this pain to characterize and describe what they feel, and it is equally hard for people who do not experience it to understand what it is like. This article has described the experience of older people living with chronic pain brought about by spine deformity, specifically in relation to the Chilean context. This research provides an in-depth understanding of the day-to-day life experiences of the subjects and how the chronic pain affects their quality of life from a cultural and contextual perspective.

The results suggest that these participants have very close ties with their children, so the family must be part of the treatment, so that the family can understand the situation they are undergoing. In addition, governments must improve the conditions of public transport, which helps people with chronic pain to move to medical care and meet with families and friends, thus improving their quality of life.

## Figures and Tables

**Table 1:** Participant characteristics.

No.	Age	Gender	Type of spinal issue	ODI score (%)	SRS-22r score
1	68	F	Spinal fracture and deformity	100	1.36
2	79	F	Spinal fracture	56	2.57
3	76	M	Spinal deviation and fracture	4	4.68
4	76	F	Spinal deformity	38	3.50
5	76	F	Multiple spine fractures	40	2.50
6	74	F	Spinal deformity	44	2.82
7	67	F	Spinal deformity, osteoporosis	49	1.90
8	76	F	Lumbar osteoarthritis	40	3.00
9	67	F	Vertebra fracture	18	4.29
10	72	M	Spinal deformity	30	3.23

## Data Availability

The interview data used to support the findings of this study have not been made available because they are restricted by the Pontificia Universidad Católica Ethics Committee in order to protect patient privacy.
